# Exposure of Triclosan in Porcine Oocyte Leads to Superoxide Production and Mitochondrial-Mediated Apoptosis during In Vitro Maturation

**DOI:** 10.3390/ijms21093050

**Published:** 2020-04-26

**Authors:** Hyo-Jin Park, Bong-Seok Song, Jin-Woo Kim, Seul-Gi Yang, Sun-Uk Kim, Deog-Bon Koo

**Affiliations:** 1 Department of Biotechnology, College of Engineering, Daegu University, 201 Daegudae-ro, Jillyang, Gyeongsan, Gyeongbuk 38453, Korea; wh10287@naver.com (H.-J.P.); 38317kjw@hanmail.net (J.-W.K.); foreverday37@naver.com (S.-G.Y.); 2 Institute of Infertility, Daegu University, 201 Daegudae-ro, Jillyang, Gyeongsan, Gyeongbuk 38453, Korea; 3 National Primate Research Center & Futuristic Animal Resource and Research Center, Korea Research Institute of Bioscience and Biotechnology, Ochang, Chungbuk 28116, Korea; sbs6401@kribb.re.kr

**Keywords:** triclosan, superoxide, Mito-TEMPO, cumulus cell expansion, apoptosis, porcine oocyte maturation

## Abstract

While triclosan (TCS) exerts detrimental effects on female reproduction, the effect of TCS-derived toxins on porcine oocytes during in vitro maturation (IVM) is unclear. This study investigated the effects of TCS on mitochondrion-derived reactive oxygen species (ROS) production and apoptosis pathways during porcine oocyte maturation. Porcine oocytes were treated with TCS (1, 10, and 100 μM) and triphenylphosphonium chloride (Mito-TEMPO; 0.1 μM), and matured cumulus oocyte complexes (COCs) were stained with orcein, dichlorofluorescein diacetate (DCF-DA), and Mito-SOX. Proteins and mRNA levels of factors related to cumulus expansion and mitochondrion-mediated apoptosis and antioxidant enzymes were analyzed by western blotting and reverse-transcription polymerase chain reaction (RT-PCR), respectively. Meiotic maturation and cumulus cell expansion significantly decreased for COCs after TCS treatment along with an increase in mitochondrial superoxide levels at 44 h of IVM. Further, mitochondrion-related antioxidant enzymes and apoptosis markers were significantly elevated in porcine COCs following TCS-mediated oxidative damage. The protective effect of Mito-TEMPO as a specific superoxide scavenger from TCS toxin improved the maturation capacity of porcine COCs. Mito-TEMPO downregulated the mitochondrial apoptosis of TCS-exposed porcine COCs by reducing superoxide level. In conclusion, our data demonstrate that TCS mediates toxicity during porcine oocyte maturation through superoxide production and mitochondrion-mediated apoptosis.

## 1. Introduction

Triclosan (TCS) is a broad-spectrum antimicrobial agent widely used in pharmaceuticals and personal-care products at concentrations of 0.1–0.3% (*w/w*) [1]. TCS has been detected in human urine, blood serum, breast milk, liver, and adipose tissue [2]. Previous studies have shown that the molecular structure of TCS is similar to that of anthropogenic estrogen, and increasing attention has been directed toward its potential toxicity to the reproduction system [1,3]. In addition, TCS may be a potential endocrine disrupter targeting the estrogen receptor (ER) [3] and is thought to impair lipid metabolism in zebrafish [4]. Although the effects of TCS have been reported in female rats [5], its impact on female fertility has not been investigated.

TCS-mediated production of reactive oxygen species (ROS) causes oxidative stress and may lead to DNA damage. TCS was found to induce oxidative stress and genotoxic response in goldfish [6], was associated with oxidative stress, and activated the antioxidant system in *Trissolcus japonicas* [7]. Further, TCS is thought to exert potential biochemical and genetic toxicities in *Eisenia fetida* [8]. In particular, it stimulates the release of superoxide radicals from the mitochondrion and interferes with mitochondrial respiration in the human epithelial cell line HaCaT [9]. These responses following TCS exposure may cause reproductive defects and embryo implantation failure in females. Many studies have shown that regulation of the mitochondrial antioxidant system is important in protecting oocytes from oxidative stress and plays an important role in oocyte maturation, follicle development, fertilization, and blastocyst development in mammals [10,11,12,13]. Although toxic effects of TCS in the form of oxidative stress and mitochondrial dysfunction have been reported, information on the effect of TCS-mediated production of mitochondrial superoxide on meiotic maturation and cumulus expansion of porcine cumulus oocyte complexes (COCs) is incomplete.

Bisphenol A (BPA) and TCS are endocrine-disrupting chemicals with structural similarity to 17β-estradiol, an estrogen hormone [14]. BPA is reported to interfere with the coordinated actions of progesterone/estrogen and impairs the receptivity of uterus and embryo migration [15]. In addition, exposure of mouse oocytes to BPA produced defects in embryo transition and uterine receptivity and caused preimplantation embryo development in mice [15]. Our previous study demonstrated the important role of BPA-induced ROS and superoxide production in mitochondrial functions and mitochondrion-mediated apoptosis activation during in vitro porcine oocyte maturation [16]. Although several studies have demonstrated the mechanism underlying the oxidative damage induced by BPA and preimplantation embryo development, the effects of TCS on oocyte maturation in pigs have not been reported.

To investigate the oxidative stress and superoxide production upon exposure to TCS, we evaluated meiotic maturation of porcine oocyte and cumulus cell expansion of porcine COCs after 44 h of in vitro maturation (IVM). We confirmed the changes in intracellular ROS and mitochondrion-derived superoxide production in porcine COCs during IVM following TCS exposure. In addition, we determined the protective effects of triphenylphosphonium chloride (Mito-TEMPO), a specific superoxide scavenger, against TCS-induced superoxide production by evaluating alterations in mitochondrion-related antioxidant enzymes and apoptosis of porcine COCs.

## 2. Results

### 2.1. Effects of TCS on Meiotic Maturation and Cumulus Expansion of Porcine Cocs

**Figure 1:**
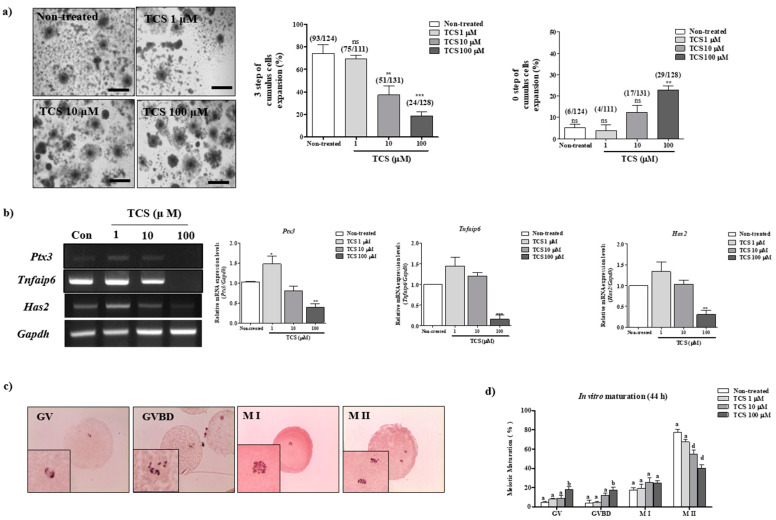
TCS-affected porcine oocyte maturation in vitro. (**a**) Changes in the percentage of cumulus cell expansion for matured porcine COCs after TCS treatment at different concentrations (1, 10, and 100 μM), as described in Supplementary Figure S1a. Scale bars = 200 µM. (**b**) The mRNA levels of cumulus cell expansion factors such as Ptx3, Tnfaip6, and Has2 in maturing porcine COCs were measured using RT-PCR. Relative fold changes in gene levels were determined after normalization to Gapdh level. Histogram values of densitometry analysis were obtained using ImageJ software. All experiments were performed in triplicates, and all values are presented as the mean ± standard error of the mean (S.E.M). Data were analyzed using one-way ANOVA, followed by Bonferroni’s multiple comparison test. Differences were considered significant at * *p* < 0.05, ** *p* < 0.01, and *** *p* < 0.001 compared with the non-treated group (**c**) Meiotic stages were classified as Gernimal vesicle (GV), GVBD, Metaphase I (M I), and M II using orcein staining. We observed the orcein stained oocytes per stage of IVM (Enlarged image, Lower left). (**d**) Diagram of meiotic maturation of porcine oocyte following TCS treatment (1, 10, and 100 µM) after 44 h, as analyzed using orcein staining. Summarized table of porcine oocyte meiotic maturation. Data are presented as the mean ± SD. Different superscript letters indicate significant differences (*p* < 0.05).

As shown in Table 1 and Figure 1a,b, we investigated the effects of different concentrations (1, 10, and 100 µM) of TCS on cumulus cell expansion for porcine COCs during IVM, as previously described [16]. As expected, changes in the morphology of cumulus cells significantly reduced (*p* < 0.001) following exposure to 100 µM TCS as compared with that reported for non-treated matured COCs. mRNA levels of the factors related to cumulus cell expansion (*Ptx3*, *Tnfaip6*, and *Has2*) were significantly lower in porcine-matured COCs treated with 10 and 100 µM TCS (*p* < 0.01 for *Has2* and *PTX3* and *p* < 0.001 for *Tnfaip6*; Figure 1b) than in non-treated COCs.

We first examined the effect of three different concentrations of TCS (1, 10, and 100 μM) on porcine oocyte maturation after 44 h of IVM. The changes in meiotic maturation were observed by staining of porcine oocytes with orcein (Table 2, Figure 1c,d). Meiotic maturation of oocytes significantly decreased when treated with 10 and 100 μM TCS in a concentration-dependent manner compared with that of non-treated oocytes (non-treated groups; 77.5% ± 6.6% vs. 1 μM TCS-treated oocytes 66.1% ± 2.7% nonsignificant difference; vs. 10 μM TCS-treated oocytes 55.1% ± 8.5%; vs. 100 μM TCS-treated oocytes, 39.8% ± 8.0%; *p* < 0.001). Thus, TCS exposure affected the in vitro meiotic maturation of porcine oocytes. These results indicate that exposure to TCS results in the inhibition of cumulus cell expansion in a TCS concentration-dependent manner.

### 2.2. Effects of TCS on ROS Production and Antioxidant Enzyme Activity of Porcine Cocs

**Figure 2:**
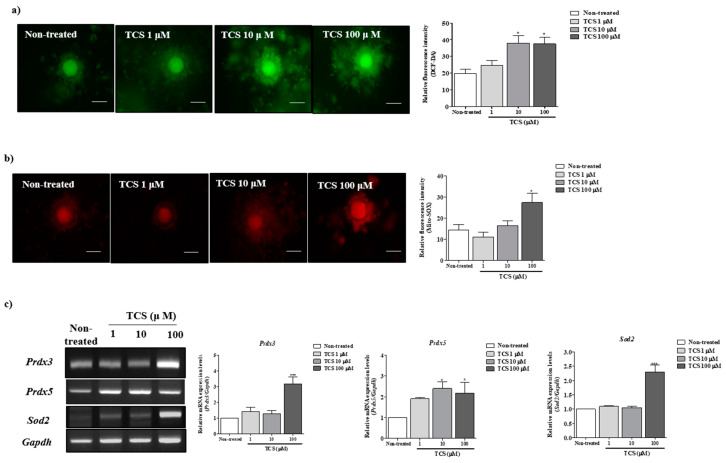
TCS induced reactive oxygen species (ROS) and superoxide production and activated mitochondrion-related antioxidant enzymes in matured porcine COCs. (**a**) Detection of intracellular ROS levels in porcine COCs after TCS treatment at different concentrations (1, 10, and 100 μM) using dichlorofluorescein diacetate (DCF-DA) (green fluorescence) staining. Scale bar = 100 µm. (**b**) Identification of mitochondrion-specific superoxides using Mito-SOX staining (red fluorescence) following exposure of COCs to TCS. Mito-SOX (red)-stained cytoplasm was examined using the iRiS™ Digital Cell Image System. Scale bar = 100 µm. (**c**) mRNA levels of mitochondrion-related antioxidant enzymes (Sod2, Prdx3, and Prdx5) in maturing porcine COCs were measured using RT-PCR, and Gapdh was used as an internal control. Histograms represent the values of densitometry analysis obtained using ImageJ software. Data in the bar graph are presented as the mean ± SEM of three independent experiments (per 30 COCs). Differences were considered significant at * *p* < 0.05, ** *p* < 0.01, and *** *p* < 0.001 compared with the non-treated group.

We investigated intracellular ROS and mitochondrial superoxide levels in porcine COCs using dichlorofluorescein diacetate (DCF-DA; green fluorescence) and Mito-SOX (red fluorescence) staining after treatment with TCS (1, 10, and 100 µM) during IVM. The green fluorescence corresponding to intracellular ROS level significantly increased in a TCS concentration-dependent manner (*p* < 0.05 for 10 and 100 µM TCS groups versus non-treated group; Figure 2a). The red fluorescence intensity of Mito-SOX as a mitochondrial ROS-specific dye was significantly higher (*p* < 0.05) for 100 µM TCS-treated porcine COCs than that reported for other groups (Figure 2b). Further, reverse-transcription polymerase chain reaction (RT-PCR) was performed to evaluate the mRNA expression of mitochondrion-related antioxidative enzymes (*Prdx3*, *Prdx5*, and *Sod2*) in porcine COCs after TCS treatment (Figure 2c). As a result, the mRNA levels of *Prdx3*, *Prdx5*, and *Sod2* were significantly higher (*Prdx3, p* < 0.05; *Prdx5, p* < 0.05; and *Sod2*, *p* < 0.001) in porcine COCs treated with 100 μM TCS than in those from other groups. Thus, porcine COCs exposed to TCS showed an increase in intracellular ROS and mitochondrial superoxide production after 44 h of IVM.

### 2.3. Effects of TCS on ROS-Derived Mitochondria Mediated Apoptosis in Porcine Cocs

**Figure 3:**
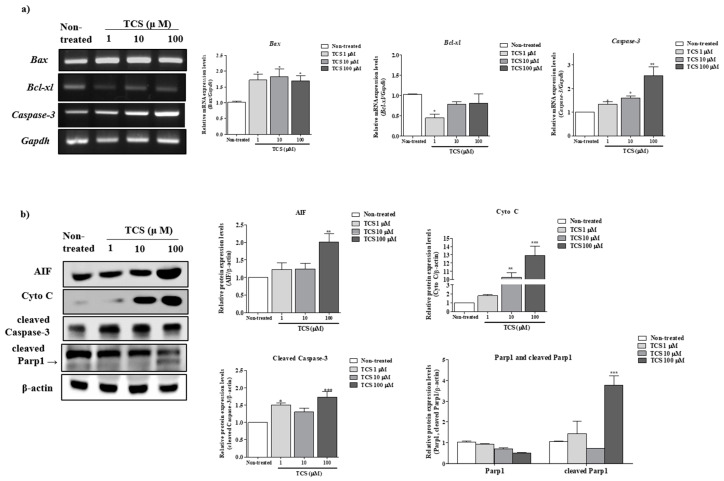
Effect of TCS exposure on mitochondrion-mediated apoptosis of porcine COCs: (**a**) The mRNA levels of mitochondrion-mediated apoptosis genes (Bax, Bcl-xl, and caspase-3) were investigated in TCS-treated COCs using RT-PCR; Gapdh served as an internal control. (**b**) Western blotting results for AIF, Cyto C, cleaved caspase-3, and cleaved PARP1 in porcine COCs. Relative fold changes in the levels of proteins related to mitochondrial apoptosis were obtained after normalization of signals to β-actin levels. Histograms represent the values of densitometry analysis obtained using ImageJ software. Data in the bar graph are presented as the mean ± SEM of three independent experiments (30 COCs). Differences were considered significant at * *p* < 0.05, ** *p* < 0.01, and *** *p* < 0.001 compared with the non-treated group.

Previous study demonstrated that the activation of apoptosis from mitochondria is associated with generation of ROS and superoxide [17]. We performed RT-PCR and Western blot analysis of mitochondrial mediated apoptosis factors (genes: *Bax, Bcl-xl*, and *Caspase-3* and proteins: Apoptosis-inducing factor (AIF), Cyto C, cleaved Caspase-3, and cleaved Parp1) in TCS-treated COCs. A significant increase of *Bax* and *Caspase-3* mRNA expression was found at 100 µM TCS for 44 h of IVM (Figure 3a). As shown in Figure 3b, compared with the non-treated group, the protein levels of AIF, Cyto C, and cleaved Caspase-3 were significantly upregulated (AIF: *p* < 0.01 and Cyto C, cleaved Caspase-3, and cleaved Parp1: *p* < 0.001) in 100 µM TCS group. Based on the results above, we selected 100 µM TCS as the exposure level for the subsequent experiment.

### 2.4. Restoration of Impaired Meiotic Maturation and Cumulus Expansion in TCS-Exposed Porcine Cocs Using Mito-TEMPO, a Specific Superoxide Scavenger

**Figure 4:**
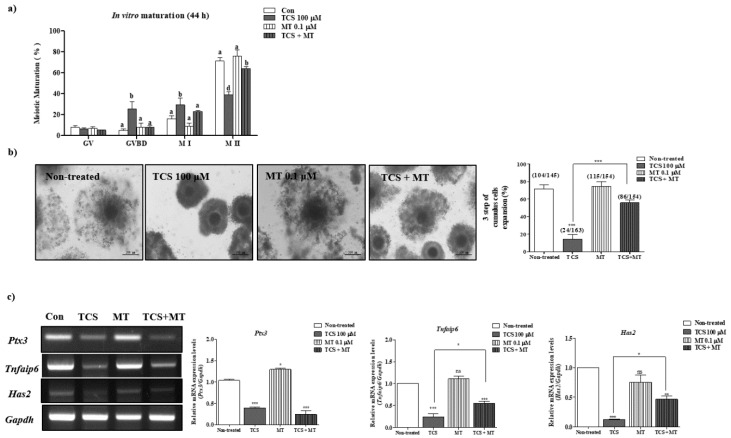
Mito-TEMPO ameliorated TCS-induced oxidative stress in porcine oocytes during in vitro maturation (IVM). Oocyte meiotic maturation was investigated by orcein staining of porcine oocytes treated with only 100 μM TCS, only 0.1 μM Mito-TEMPO (MT), and Mito-TEMPO after TCS pretreatment (TCS + MT) (Supplementary Figure S1b). (**a**) Summarized table of porcine oocyte meiotic maturation after TCS and/or Mito-TEMPO treatment. Data are presented as the mean ± SD. Different superscript letters indicate significant differences (*p* < 0.05). (**b**) Changes in cumulus cell expansion percentage in matured porcine COCs following TCS- and/or Mito-TEMPO treatment. Scale bars = 200 µM. (**c**) mRNA levels of cumulus cell expansion factors such as Ptx3, Tnfaip6, and Has2 in maturing porcine COCs after Mito-TEMPO and/or TCS treatment (non-treated, only TCS, only Mito-TEMPO: MT and TCS + MT) were measured using RT-PCR. Relative fold changes in the levels of genes were detected after normalization to Gapdh level. Data were analyzed using one-way ANOVA followed by Bonferroni’s multiple comparison test. Differences were considered significant at * *p* < 0.05, ** *p* < 0.01, and *** *p* < 0.001 compared with the control group.

Previous studies have highlighted the protective effects of Mito-TEMPO (0.1 μM) as a superoxide scavenger against BPA-induced ROS, as evident from the improvement in oocyte maturation and blastocyst development in pigs [16,18]. To evaluate the protective effects of Mito-TEMPO against TCS-induced impairment of porcine oocyte maturation, we investigated changes in meiotic maturation and cumulus cell expansion in Mito-TEMPO-treated COCs subjected to TCS pretreatment. As shown in Table 3 and Figure 4a, the TCS-mediated decrease in meiotic maturation was ameliorated in porcine COCs exposed to 0.1 μM of Mito-TEMPO (non-treated group, 74.5% ± 3.0%; 100 μM TCS group, 38.8% ± 5.2% *p* < 0.001; 0.1 μM Mito-TEMPO group, 76.2% ± 9.7%; TCS + Mito-TEMPO group, 63.8% ± 1.3%; nonsignificant difference). The reduction in cumulus expansion following TCS treatment was also rescued after exposure to 0.1 µM Mito-TEMPO (only TCS versus TCS + Mito-TEMPO, *p* < 0.001; Table 4 and Figure 4b,c).

### 2.5. TCS-Induced ROS and Superoxide Production in Porcine Cocs Was Reduced by the Protective Effect of Mito-TEMPO

**Figure 5:**
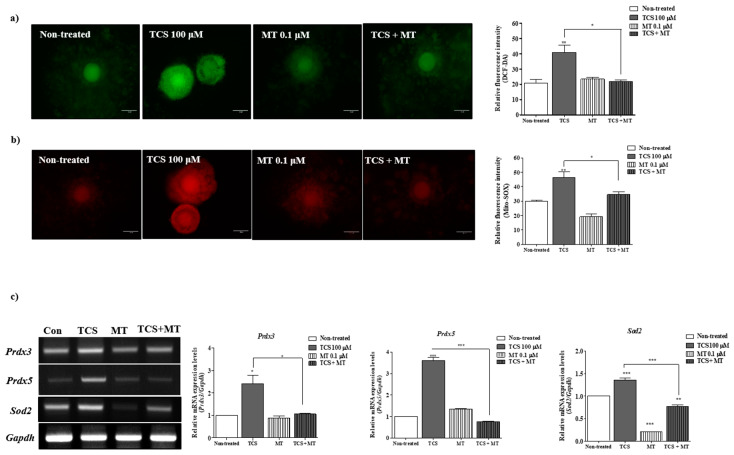
The antioxidative property of Mito-TEMPO protected porcine COCs from TCS-induced oxidative stress and superoxide production. (**a**,**b**) Detection of intracellular ROS levels and mitochondrion-specific superoxides by Mito-SOX and DCF-DA staining in porcine COCs treated with TCS (100 μM) and/or Mito-TEMPO (MT; 0.1 μM) using the iRiS™ Digital Cell Image System. Scale bar = 100 µm. Histograms represent the values of densitometry analysis obtained using ImageJ software. Data in the bar graph are presented as the mean ± SEM of three independent experiments (per 30 COCs). Differences were considered significant at * *p* < 0.05, ** *p* < 0.01, and *** *p* < 0.001 compared with the control group. (**c**) mRNA levels of mitochondrion-related antioxidant enzymes (Sod2, Prdx3, and Prdx5) in maturing porcine COCs after TCS (100 μM) and/or MT (0.1 μM) treatment were measured using RT-PCR, and Gapdh was used as an internal control. Histograms represent the values of densitometry analysis obtained using ImageJ software. Data in the bar graph are presented as the mean ± SEM of three independent experiments (per 30 COCs). Differences were considered significant at * *p* < 0.05, ** *p* < 0.01, and *** *p* < 0.001 compared with the non-treated group.

The results observed for TCS-induced ROS production using DCF-DA and Mito-SOX staining highlighted the protective effects of 0.1 μM Mito-TEMPO treatment on porcine oocyte maturation. In comparison with TCS-treated COCs, those treated with Mito-TEMP (0.1 µM) following TCS pretreatment showed significant amelioration in intracellular ROS and superoxide production (*p* < 0.05) (Figure 5a,b). In case of Mito-TEMPO-treated porcine COCs following TCS pretreatment, the expression levels of *Prdx3*, *Prdx5*, and *Sod2* mRNAs were significantly recovered (*Prdx3*: *p* < 0.05; *Prdx5* and *Sod2: p* < 0.001) (Figure 5c). These results suggest that the antioxidative effects of Mito-TEMPO regulated the TCS-induced mitochondrial ROS production and improved porcine oocyte maturation during IVM.

### 2.6. Mito-TEMPO Inhibited the Mitochondrion-Mediated Apoptosis Induced by TCS in Porcine Cocs during IVM

**Figure 6:**
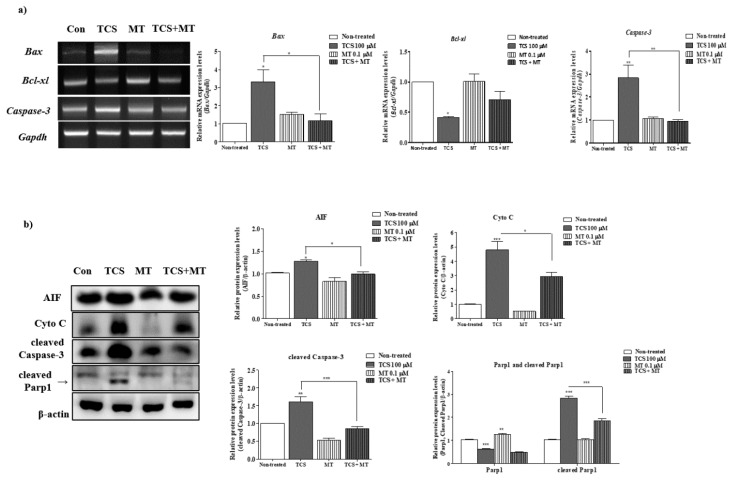
TCS-mediated mitochondrial apoptosis of porcine COCs was ameliorated by Mito-TEMPO. (**a**) The mRNA levels of mitochondrion-mediated apoptosis genes (Bax, Bcl-xl, and caspase-3) were investigated in TCS or Mito-TEMPO-treated COCs using RT-PCR. Gapdh was used as an internal control. (**b**) Western blotting results for AIF, Cyto C, cleaved caspase-3, and cleaved PARP1 in porcine COCs after TCS and/or Mito-TEMPO treatment: Relative fold changes in the levels of proteins related to mitochondrial apoptosis were calculated following normalization to β-actin level. Histograms represent the values of densitometry analysis obtained using ImageJ software. Data in the bar graph are presented as the mean ± SEM of three independent experiments (per 30 COCs). Differences were considered significant at * *p* < 0.05, ** *p* < 0.01, and *** *p* < 0.001 as compared to the non-treated group.

To confirm the protective effect of Mito-TEMPO against TCS-mediated damage, porcine COCs were exposed to 0.1 µM Mito-TEMPO after TCS pretreatment and the activities of mitochondrion-derived apoptosis pathways were determined by RT-PCR and western blot analyses. As shown in Figure 6a, the mRNA levels of *Bax* following Mito-TEMPO treatment (TCS + Mito-TEMPO) significantly reduced (*p* < 0.05; compared to only TCS treated group) in porcine COCs. Mito-TEMPO treatment significantly increased (*p* < 0.01) the mRNA expression of *Bcl-xl* (Figure 6a). Further, the mRNA level of *caspase-3* significantly decreased (*p* < 0.01) in the Mito-TEMPO-treated group as compared with that observed in only TCS-treated group. As expected, the protein levels of mitochondrion-mediated apoptosis factors AIF and Cyto C increased in porcine COCs exposed to TCS, and these levels were significantly restored (*p* < 0.05; compared to only TCS-treated group) after Mito-TEMPO treatment. Western blotting data revealed that the increased levels of cleaved caspase-3 and cleaved PARP1 in TCS-treated COCs were dramatically downregulated (*p* < 0.001) by Mito-TEMPO treatment during IVM II. Thus, the increase in mitochondrion-mediated apoptosis following TCS-induced damage was ameliorated by Mito-TEMPO during the IVM of porcine oocytes.

## 3. Discussion

This is the first study to elucidate the toxicological effects of TCS on meiotic maturation and cumulus cell expansion in porcine oocytes during IVM through evaluation of oxidative stress and mitochondrion-specific superoxide (O_2_^−^) production. We found that the treatment of porcine COCs with a relatively high concentration of TCS (100 μM) resulted in a significant decrease in meiotic maturation and cumulus cell expansion (Figure 1 and Table 2 and Table 3). This observation was consistent with the significant increase in intracellular ROS and mitochondrion-specific superoxide production in a TCS dose-dependent manner (Figure 2).

TCS is a common ingredient of several personal-care products such as shampoo, toothpaste and soaps, textiles, and infant toys [19]. In previous study, the daily of human exposure to TCS exposure concentration varies between 0.047 and 0.073 mg/kg/day [20]. TCS is known as an endocrine-disrupting chemical and is widely detected in the human urine, blood, and breast milk [3,21]. TCS is not acutely toxic to mammals, but previous studies have suggested the estrogenic properties and endocrine-disrupting effects of TCS [3]. The exposure of female yellow river carp to TCS was found to enhance the synthesis and secretion of ER and active 17β-estradiol (E2) via ER-mediated and non-ER-mediated pathways [22]. While E2 had no effect on meiotic maturation, it significantly decreased germinal vesicle breakdown (GVBD) percentage in the presence of follicle-stimulating hormone (FSH). This result suggested that E2 may have a potential differing role in oocyte maturation as a part of an intricate system during follicular development in vivo [23]. On the other hand, a previous study suggested that the transient E2 supplementation could improve the metaphase II (M II) oocyte production rate and further blastocyst developmental competence in pigs [24]. Thus, estrogen may be necessary for improving meiotic maturation during IVM. However, the toxic effects of TCS on oocyte maturation and cumulus cell expansion-mediated via oxidative stress or superoxide production in the female reproduction system are poorly understood. Therefore, the purpose of the present study was to characterize the toxicological influence of TCS on the IVM of porcine oocytes by the analysis of mitochondrion-related superoxide production.

The enzymatic antioxidant system plays a crucial role in the elimination of ROS to maintain the balance between its production and removal [25]. In addition, ROS generation from embryo metabolism and oocyte maturation has been confirmed via various antioxidative enzymatic mechanisms in bovine [26], pigs [27], and humans [28]. As shown in Figure 2, TCS induced the expression of mRNAs related to the production of mitochondrial ROS and mitochondrion-related antioxidative enzymes in porcine COC after IVM. TCS potentiates the cytotoxicity of hydrogen peroxide (H_2_O_2_) and may induce oxidative stress in rat thymocytes [29]. As one of the antioxidative enzymes in the mitochondria, superoxide dismutase may prevent the accumulation of superoxides [30]. The mitochondrial antioxidant system, including peroxiredoxin (Prdx) 3, Prdx5, and Mn SOD, is modulated by the mitochondrion-derived ROS [31]. In the current study, the transcripts levels of mitochondrion-related antioxidant enzymes (*Sod2, Prdx3*, and *Prdx5*) and Mito-SOX staining intensity increased following treatment of porcine COCs with TCS during IVM. These results indicate that TCS may disrupt the mitochondrion-related antioxidant system during porcine oocyte maturation.

We observed an increase in the mitochondrial apoptosis rate via superoxide production in TCS-exposed porcine COCs (Figure 3). The detrimental effects of TCS on mitochondrion-related mechanisms have been previously reported. Evaluation of rat liver mitochondria exposed to TCS revealed impaired oxidative phosphorylation, reduced ATP synthesis, and excessive oxygen uptake [32]. Short exposure to low concentrations of TCS paralyzed the progressive motility of spermatozoa [32]. Phagocyte NAD(P)H oxidase inhibitors could induce mitochondrion-mediated apoptosis pathways through mitochondrial superoxides [33]. Excessive accumulation of superoxide and ROS interferes with the maturation of the nucleus and cytoplasm, consequently inducing cellular apoptosis. In our results, TCS-induced mitochondrial superoxide production in matured porcine COCs activated the signaling pathways related to mitochondrion-mediated apoptosis. Based on these results, we conclude that TCS exposure exerted negative effects on meiotic maturation or cumulus cell expansion through oxidative stress and mitochondrial apoptosis.

Mito-TEMPO is a specific mitochondrial superoxide scavenger that mimics the activity of superoxide dismutase [34]. Our previous study demonstrated the positive effects of Mito-TEMPO on porcine embryo development that were mediated by improving mitochondrial functions [18] and by protecting BPA-induced oxidative stress during porcine oocyte maturation [16]. Although the protective effects of Mito-TEMPO against BPA toxicity on meiotic maturation and cumulus cell expansion have been investigated, its recovery effects as a superoxide scavenger on ROS produced after TCS exposure during porcine oocyte maturation have not been reported. We speculated that the toxic effects of TCS were mediated through the generation of excessive ROS and mitochondrial superoxides; this may induce oxidative stress, as observed with BPA. As expected, the TCS-exposed oocytes showed defects in maturation capacity owing to increased superoxide and ROS production, and these effects were ameliorated by Mito-TEMPO (Figure 5 and Table 4 and Table 5).

**Figure 7:**
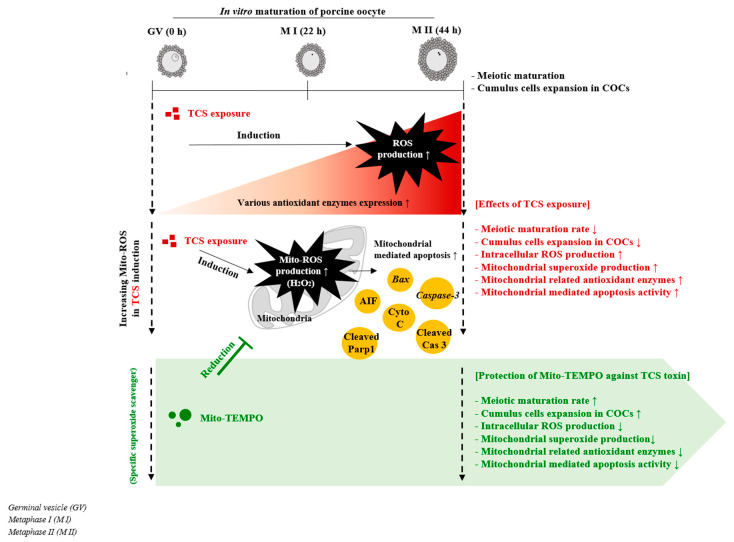
Protective effects of Mito-TEMPO against TCS-induced superoxide production on porcine oocyte maturation: TCS-derived superoxides affected the meiotic maturation of porcine oocytes via mitochondrial apoptosis in vitro. Graphical summary. Red part: Mitochondrial ROS production and oxidative stress in response to TCS exposure during porcine oocyte maturation resulted in a decrease in meiotic maturation and cumulus cell expansion and an increase in mitochondrion-mediated apoptosis in maturing porcine COCs. Green part: The toxic effects of TCS-induced oxidative stress during the IVM of porcine oocytes were recovered by the protective functions of Mito-TEMPO.

Studies with mouse models have suggested that exposure to BPA during preimplantation may lead to implantation failure and preimplantation embryo development [15]. Moreover, a positive association was reported between BPA exposure and implantation failure in patients undergoing in vitro fertilization [2]. Overall, our findings are similar to the previously reported negative effects of BPA-induced mitochondrial ROS production and mitochondrion-mediated apoptosis on porcine oocyte maturation and cumulus cells [16]. TCS-exposed COCs showed an increase in the level of mitochondrion-mediated apoptosis. As shown in Figure 6, RT-PCR and western blotting showed similar results related to the toxicity of BPA on porcine oocytes, as previously described [16]. However, the correlation between TCS exposure and female infertility via dysfunction or defects of mitochondrion-related mechanism is still unknown. Furthermore, whether TCS-mediated mitochondrial superoxide production, induction of oxidative stress, and mitochondrial apoptosis affect implantation or fertilization capacity during IVM and *in vitro* fertilization (IVF) has not been investigated. Our results provide valuable information that porcine oocyte maturation during IVM was adversely affected with TCS exposure via the mitochondrial superoxide production. However, in the study, the Triclosan concentration of 100 uM was never physiologically observed in the body, although to date, there are insufficient reports of detection of TCS in female reproduction system such as ovaries, uterus, and vagina. The present study demonstrates that TCS exposure exerts a negative impact on the meiotic maturation and cumulus expansion of porcine COCs during IVM and alters mitochondrial superoxide-induced apoptosis activity (Figure 7, graphical summary). Therefore, TCS-induced superoxide production affects porcine oocyte maturation and cumulus cell expansion by damaging the mitochondrial antioxidant enzyme system.

## 4. Materials and Methods

### 4.1. Chemicals

All chemicals used in this study were purchased from Sigma Chemical Co. (St. Louis, MO, USA), unless otherwise stated.

### 4.2. IVM

Porcine ovaries were obtained from 6-month-old female pigs (Yorkshire/Landrace (♀) × Duroc (♂), 100 kg) from local abattoirs (Gyeongsan and Daegu City) and transported to the laboratory in 0.9% saline (*w*/*v*) supplemented with 75 μg/mL potassium penicillin G at around 30–35 °C. Immature COCs were aspirated from follicles (3–6 mm in diameter) using a 10-mL syringe and an 18-gauge needle. The undamaged COCs with similar quality of cytoplasm and surrounding cumulus cells were selected by mouth pipetting and washed thrice with Tyrode’s lactate-N-2-hydroxyethylpiperazine-N′-2-ethanesulfonic acid (TL-HEPES) medium. Immature COCs were selected under an optical microscope, as previously described [16]. Approximately 50–60 immature COCs were subjected to maturation in 500 µL of IVM medium at 38.5 °C and 5% CO_2_. North Carolina State University-23 (NCSU-23) medium supplemented with 10% follicular fluid (*v*/*v*), 0.57 mM cysteine, 10 ng/mL β-mercaptoethanol, 10 ng/mL epidermal growth factor, 10 IU/mL pregnant mare’s serum gonadotropin (PMSG), and 10 IU/mL human chorionic gonadotropin (hCG) was used for oocyte maturation. After cultivation for 22 h (IVM I), the oocytes were matured in the same medium without PMSG/hCG for 22 to 44 h (IVM II). TCS was prepared in dimethyl sulfoxide (DMSO). During maturation, the cells were exposed to TCS (concentrations of 1, 10, and 100 µM) added to the maturation medium NCSU-23 for 44 h as previously described [35]. Mito-TEMPO (0.1 µM) was added during IVM II to reduce TCS-induced mitochondrial ROS production following pretreatment with 100 µM TCS for 22 h (Supplementary Figure S1).

### 4.3. Assessment of Cumulus Cell Expansion and Acetic-Orcein Staining

Cumulus expansion in porcine COCs was evaluated under a microscope (Leica, Solms, Germany) after 44 h of IVM in the presence or absence of TCS and Mito-TEMPO. The process was divided into three steps as previously described [16,36]. After 44 h, meiotic maturation was distinguished based on nuclear stages. Oocytes denuded by pipetting in TL-HEPES medium supplemented with 0.1% hyaluronidase were rinsed with 0.1% polyvinyl alcohol (PVA) in phosphate-buffered saline (PVA-PBS) and mounted on microscope slides. The samples were fixed for 3 days in acetic acid/ethanol (1:3, *v/v*) and then stained with 1% acetic-orcein (*v/v*) for 5 min. Meiotic stages of samples were investigated under a microscope (Leica, Solms, Germany).

### 4.4. RNA Extraction and RT-PCR

Total RNA was isolated using the Trizol reagent (Invitrogen, Carlsbad, CA, USA) according to the manufacturer’s instructions. RNA concentration and purity were measured using OPTIZEN NanoQ (Mecasys, Daejeon, Korea), and 1 µg/µL of total RNA was used to synthesize cDNA using AccuPower^®^ RT-PCR Premix (Bioneer Inc., Daejeon, Korea). Specific primers for the sequences of interest (Table 5) were designed using the National Center for Biotechnology Information (NCBI) database. PCR was performed as follows: 95 °C for 5 min, followed by 30–35 cycles at 95 °C for 30 s, 55 to 61 °C for 30 s, 72 °C for 30 s, and 72 °C for 5 min. The PCR products were separated by electrophoresis on a 2% agarose gel, stained with ethidium bromide, and photographed under UV illumination. Band intensities were quantified using ImageJ (National Institutes of Health, MD, USA).

### 4.5. DCF-DA and Mito-SOX Staining

The levels of intracellular ROS in porcine COCs were measured using the DCF-DA method (Molecular Probes, Eugene, OR, USA), as previously described [37]. After washing thrice with PVA-PBS medium, mature COCs were incubated in IVM II medium containing 5 μM DCF-DA for 20 min at 38.5 °C and 5% CO_2_. Following incubation, the COCs were washed thrice with 0.1% PVA-PBS, and their fluorescence was recorded using an Olympus DP 70 camera (Olympus, Tokyo, Japan). Mito-SOX (red)-stained cytoplasm was examined using the iRiS™ Digital Cell Image System (Logos Biosystems, Inc., Anyang, Korea). Porcine COCs were washed thrice in PBS-PVA and cultured in 300 μL of IVM II medium mixed with Mito-SOX (500:1, Invitrogen) at 37 °C for 30 min. The COCs were then fixed with 2.5% glutaraldehyde/PBS (*v/v*) for 1 h and permeabilized with 0.1% Triton X-100 (*v/v*) for 30 min, followed by staining with Hoechst 33342 (2 mg/mL). The COCs were covered with a cover glass after mounting on glass slides using a mounting solution.

### 4.6. Protein Extraction and Western Blot Analysis

Matured COCs (30 per sample) were lysed using PRO-PREP protein lysis buffer (iNtRON, Daejeon, Korea), and the lysates were separated on 12% sodium dodecyl sulfate polyacrylamide gel electrophoresis (SDS-PAGE) gels. The separated protein bands were transferred onto nitrocellulose membranes (Pall Life Sciences, Port Washington, NY, USA), and the membranes were incubated with anti-cleaved caspase-3 (1:3000; #9664; Cell Signaling, MA, USA), anti-PARP1/cleaved PARP1 (1:4000; Santa Cruz, CA, USA), anti-AIF (1:3000; Santa Cruz), anti-Cyto C (Cyto C; 1:3000; Abcam, Cambridge, MA, USA), and anti-β-actin (1:3000; Santa Cruz) antibodies. The membranes were then probed with a secondary horseradish peroxidase (HRP)-conjugated anti-mouse/rabbit IgG (Thermo, Rockford, IL, USA) or an anti-goat IgG (AbFrontier, Seoul, Korea) secondary antibody overnight. Blots were developed using an enhanced chemiluminescence (ECL) kit (Advansta, Menlo Park, CA, USA) according to the manufacturer’s instructions. The amount of protein was calculated depending on band densities using Fusion Solo software (Vilber Lourmat, Collégien, France), and the bands were scanned using ImageJ software (NIH).

### 4.7. Statistical Analysis

All percentage data and data sets were subjected to arcsine transformation and expressed as the mean ± standard deviation (SD). All values from western blot and RT-PCR experiments are presented as the mean ± standard error of the mean (SEM). The results were analyzed using one-way analysis of variance (ANOVA) followed by Bonferroni’s and Tukey’s multiple comparison test and *t*-test. Histograms for densitometry analysis were obtained using ImageJ (NIH). All data were plotted using GraphPad Prism 5.0 (San Diego, CA, USA). Differences were considered significant at * *p* < 0.05, ** *p* < 0.01, and *** *p* < 0.001.

## 5. Conclusions

In summary, our study provides the first evidence of the toxic effects of TCS on porcine oocyte maturation and cumulus cells in vitro. This report not only reveals the mechanism underlying the toxic effects of TCS on matured porcine COCs after IVM but also highlights the preventive effects of Mito-TEMPO against TCS-induced damage to oocytes.

## Figures and Tables

**Table 1:** Effect of triclosan (TCS) treatment on cumulus cells expansion patterns of porcine cumulus oocyte complexes (COCs).

TCS (μM)	No. of COCs Examined	% of Cumulus Cells Expansion (*n*)			
		3 step	2 step	1 step	0 step
Non-treated	130	72.1 ± 6.6 (93) ^a^	12.2 ± 4.1 (16)	8.9 ± 4.1 (12)	6.8 ± 1.4 (9) ^a^
1	111	69.1 ± 5.9 (75) ^a^	21.7 ± 10.7 (27)	5.5 ± 5.3 (5)	3.8 ± 5.0 (4) ^a^
10	134	35.9 ± 5.8 (49) ^c^	40.6 ± 11.1 (52)	11.7 ± 5.5 (16)	11.9 ± 5.1 (17) ^a^
100	146	11.5 ± 0.9 (17) ^d^	26.8 ± 6.4 (38)	30.1 ± 2.8 (44)	31.5 ± 5.3 (47) ^c^

Data are expressed as means ± SD of three independent experiments. Different superscript letters denote significant differences (*p* < 0.05).

**Table 2:** Effect of TCS treatment on meiotic maturation of porcine oocytes.

TCS (μM)	No. of Oocytes Examined	% of Oocytes (*n*)			
		GV	GVBD	M I	M II
Non-treated	142	4.3 ± 3.3 (5) ^a^	4.0 ± 6.5 (4) ^a^	17.7 ± 4.6 (24) ^a^	77.5 ± 6.6 (109) ^a^
1	136	8.1 ± 0.8 (11) ^a^	4.6 ± 1.6 (6) ^a^	19.1 ± 5.3 (27) ^a^	66.1 ± 2.7 (92) ^a^
10	149	9.1 ± 5.0 (12) ^a^	11.8 ± 5.4 (16) ^a^	25.2 ± 10.7 (38) ^a^	55.1 ± 8.5 (83) ^d^
100	151	18.3 ± 5.3 (28) ^b^	17.2 ± 6.2 (25) ^b^	24.6 ± 5.6 (36) ^a^	39.8 ± 8.0 (62) ^d^

Data are expressed as means ± SD of three independent experiments. Different superscript letters denote significant differences (*p* < 0.05).

**Table 3:** Changes in meiotic maturation rate in porcine COCs of TCS and/or Mito-TEMPO treated groups.

TCS (100 μM)	Mito-TEMPO (0.1 μM)	No. of Oocytes Examined	% of Oocytes (*n*)			
			GV	GVBD	M I	M II
-	-	110	7.8 ± 3.2 (8) ^a^	5.0 ± 2.5 (5) ^a^	16.0 ± 5.2 (18) ^a^	74.5 ± 3.0 (79) ^a^
+	-	139	6.4 ± 1.8 (9) ^a^	25.6 ± 11.6 (36) ^b^	29.3 ± 11.8 (40) ^c^	38.8 ± 5.2 (54) ^d^
-	+	106	6.8 ± 2.8 (8) ^a^	7.9 ± 7.0 (10) ^a^	9.0 ± 4.8 (10) ^a^	76.2 ± 9.7 (78) ^a^
+	+	134	5.2 ± 0.4 (7) ^a^	7.8 ± 2.8 (11) ^b^	23.1 ± 1.5 (31) ^b^	63.8 ± 1.3 (85) ^a^

Data are expressed as means ± SD of three independent experiments. Different superscript letters denote significant differences (*p* < 0.05).

**Table 4:** Changes in cumulus cells expansion patterns in porcine COCs of TCS and/or Mito-TEMPO-treated groups.

TCS (100 μM)	Mito-TEMPO (0.1 μM)	No. of COCs Examined	% of Cumulus Cells Expansion (*n*)			
			3 step	2 step	1 step	0 step
-	-	145	71.8 ± 8.2 (104) ^a^	13.7 ± 5.0 (25) ^a^	9.7 ± 6.1 (18) ^a^	4.8 ± 2.4 (11) ^a^
+	-	163	14.5 ± 8.6 (24) ^d^	27.1 ± 4.7 (54) ^b^	24.6 ± 4.9 (49) ^b^	33.8 ± 5.7 (69) ^d^
-	+	154	74.7 ± 9.1 (115) ^a^	8.9 ± 2.4 (17) ^a^	10.1 ± 7.5 (18) ^a^	5.7 ± 4.8 (11) ^a^
+	+	154	55.7 ± 5.3 (86) ^a^	15.6 ± 3.8 (30) ^a^	15.0 ± 4.8 (27) ^a^	13.6 ± 2.9 (29) ^a^

Data are expressed as means ± SD of three independent experiments. Different superscript letters denote significant differences (*p* < 0.05).

**Table 5:** Primer sequences of cumulus expansion factors, mitochondria-related antioxidant enzymes, and apoptosis factors from matured COCs for RT-PCR.

Genes	Primer Sequences	Tm °C	Gene Bank Accession No.	Base Pairs
*Has2*	F(5′–3′): TGGCTGTACAATGCGATGTG R(5′–3′): TGGGTGGTGTGATTTTCACC	55	(NM_214053.1)	402
*Tnfaip6*	F(5′–3′): TCTTCCTGTGGGAAGAGGCT R(5′–3′): GTCCGTCTGAACAGAAGCGA	55	(NM_001159607.1)	337
*Ptx3*	F(5′–3′): TCAGTGCCTGCATTTGGGTC R(5′–3′): TTCTGAACAAGGGCATGTAG	58	(NM_001244783.1)	225
*Sod2*	F(5′–3′): GCAGCTCGAGCAGGAATCTGG R(5′–3′): ACGCGGCCTACGTGAACAA	59.7	(NM_214127.2)	163
*Prdx3*	F(5′–3′): AGTGGATTCCCACTTCAGCC R(5′–3′): AACCCATGGAGAAGTCTGCC	55.1	(NM_001244531.1)	290
*Prdx5*	F(5′–3′): ACCTTCCAGGGTTTGTGGAG R(5′–3′): CCTGAATGTGGAGCCAGATG	55	(NM_214144.1)	285
*Bax*	F(5′–3′): AAGCGCATTGGAGATGAACT R(5′–3′): CTGGACTTCCTTCGAGATCG	50	(XM_003127290.4)	251
*Bcl-xl*	F(5′–3′): AGGGCATTCAGTGACCTGAC R(5′–3′): CACCTAGAGCCTTGGATCCA	55	(NM_214285.1)	242
*Caspase3*	F(5′–3′): GAGGCAGACTTCTTGTATGC R(5′–3′): TTCCATGTATTGTGTCCATGC	50	(NM_214131.1)	238
*Gapdh*	F(5′–3′): TCGGAGTGAACGGATTTC R(5′–3′): CCTGGAAGATGGTGATGG	53.7	(NM_001206359.1)	230

Has2; *HA synthase 2,* Tnfaip6; *TNF-a-induced protein 6, Ptx3; Pentraxin 3, Sod2; Superoxide dismutase 2,* Prdx3; Peroxiredoxin 3, Prdx5; Peroxiredoxin 5, Bax; Bcl2-associated X protein, Bcl-xl; *B-cell lymphoma-extra large*, *Gapdh; Glyceraldehyde-3-Phosphate Dehydrogenase, Tm; Meting* Temperature, F; Forward, R; Reverse.

## Supplementary Materials

The following are available online at https://www.mdpi.com/1422-0067/21/9/3050/s1.

## Abbreviations

triclosan	(TCS)
in vitro maturation	(IVM)
reactive oxygen species	(ROS)
triphenylphosphonium chloride	(Mito-TEMPO)
cumulus oocyte complexes	(COCs)
dichlorofluorescein diacetate	(DCF-DA)
reverse-transcription polymerase chain reaction	(RT-PCR)
estrogen receptor	(ER)
Bisphenol A	(BPA)
17β-estradiol	(E2)
germinal vesicle breakdown	(GVBD)
follicle-stimulating hormone	(FSH)
metaphase II	(M II)
hydrogen peroxide	(H_2_O_2_)
Tyrode’s lactate-N-2-hydroxyethylpiperazine-N’-2-ethanesulfonic acid	(TL-HEPES)
North Carolina State University-23	(NCSU-23)
pregnant mare’s serum gonadotropin	(PMSG)
human chorionic gonadotropin	(hCG)
dimethyl sulfoxide	(DMSO)
polyvinyl alcohol	(PVA)
phosphate-buffered saline	(PBS)
sodium dodecyl sulfate polyacrylamide gel electrophoresis	(SDS-PAGE)
enhanced chemiluminescence	(ECL)
one-way analysis of variance	(ANOVA)
standard deviation	(SD)
standard error of the mean	(SEM)
