# Supplementary material for: Exposure of Triclosan in Porcine Oocyte Leads to Superoxide Production and Mitochondrial-Mediated Apoptosis during In Vitro Maturation
Source: Int J Mol Sci. 2020 Apr 26;21(9):3050. doi: 10.3390/ijms21093050 (PMC7246582; doi:10.3390/ijms21093050)
Supplement: Supplementary file 1 [file ijms-21-03050-s001.pdf]

a) Experimental methods of TCS treatments (Fig.1 – 3)

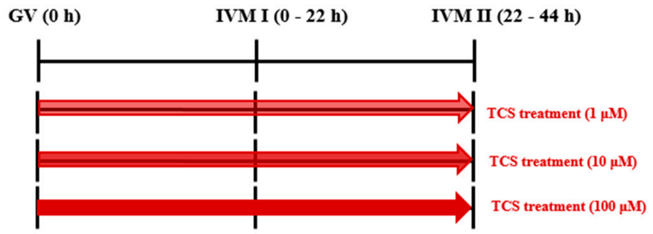

b) Recovery experiment: treatment conditions (Fig. 4)

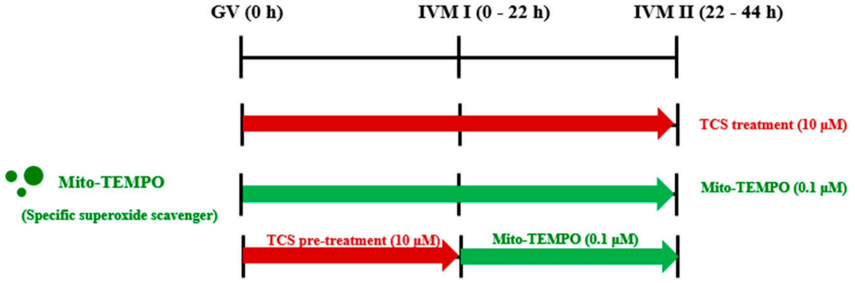

Supplementary Figure 1 Graphical description of TCS and/or Mito-TEMPO treatment in porcine oocyte maturation
